# Persistent Oropharyngeal Hemangioma Causing Progressive Upper Airway Compromise: Diagnostic and Therapeutic Challenges

**DOI:** 10.7759/cureus.113471

**Published:** 2026-07-27

**Authors:** Maria Claudia Rodríguez Duplat, Abel Enrique Manjarres Guevara, Margarita Maria Martinez Manjarres, Maria José Lara Carvajal, Natalia Gomez Acosta

**Affiliations:** 1 Department of Otolaryngology, Hospital Infantil Universitario de San José, Bogotá, COL; 2 Department of Otolaryngology, Universidad FUCS, Bogotá, COL

**Keywords:** oropharyngeal hemangioma, propranolol, soft palate, upper airway obstruction, vascular anomaly

## Abstract

Hemangiomas are rare benign vascular tumors of the oral cavity. Lesions involving the soft palate and oropharynx may cause significant functional impairment and, in advanced cases, life-threatening upper airway compromise. We report the case of a 15-year-old girl with a longstanding palatal mass first identified in early childhood who presented with progressive enlargement of the lesion, accompanied by dyspnea, odynophagia, and globus sensation. Physical examination and flexible nasolaryngoscopy revealed an extensive violaceous vascular lesion causing partial upper airway obstruction. Contrast-enhanced computed tomography (CT) and CT angiography demonstrated a hypervascular lesion consistent with a probable clinicoradiologic diagnosis of hemangioma, without evidence of osseous involvement. Given the lesion's extensive size and anatomically challenging location, surgical resection was deemed unsuitable, and oral propranolol was initiated following multidisciplinary evaluation. During six months of clinical follow-up, the patient demonstrated resolution of respiratory symptoms, no treatment-related adverse effects, and apparent clinical improvement. This case highlights the importance of a comprehensive diagnostic evaluation and individualized multidisciplinary management for complex vascular lesions involving the upper airway.

## Introduction

The 2025 International Society for the Study of Vascular Anomalies (ISSVA) classification defines hemangiomas as benign vascular tumors characterized by active endothelial cell proliferation [1,2]. Although their pathogenesis has yet to be fully elucidated, growing evidence supports a multifactorial process driven by dysregulated vasculogenesis and angiogenesis. Among the proposed mechanisms, placental embolization and tissue hypoxia have received particular attention, supported by the identification of placental-specific endothelial markers within infantile hemangioma tissue [3,4].

Hemangiomas account for approximately 7-10% of tumors diagnosed during infancy. More than half occur within the head and neck region, whereas involvement of the oral cavity remains distinctly uncommon. These tumors exhibit a marked female predominance, with an approximate female-to-male ratio of 3:1. In addition, up to 20% of affected patients present with multiple cutaneous hemangiomas, a finding that should prompt evaluation for associated visceral lesions [5,6].

Hemangiomas involving the soft palate or oropharynx may produce both respiratory and alimentary tract obstruction, creating substantial diagnostic and therapeutic challenges. Accurate assessment relies on multimodal imaging, including contrast-enhanced computed tomography, CT angiography, and flexible nasolaryngoscopy, which together allow precise delineation of lesion extent, vascular anatomy, and the degree of functional compromise [2,7].

Since its unexpected therapeutic effect was first recognized in 2008, propranolol, a nonselective β-adrenergic antagonist, has revolutionized the management of complicated infantile hemangiomas and is currently regarded as the first-line treatment for infants with proliferating infantile hemangiomas requiring systemic therapy. Its clinical efficacy is attributed to a combination of immediate vasoconstriction, inhibition of angiogenesis, and promotion of endothelial cell apoptosis. The U.S. Food and Drug Administration (FDA) recommends an initial dosage of 0.6 mg/kg twice daily, with escalation to a target dosage of 1.7 mg/kg twice daily, while maintenance doses of 2-3 mg/kg/day are supported by substantial clinical evidence [7,8]. Although these recommendations were established for infantile hemangiomas during infancy, propranolol has also been used on an individualized basis in selected older patients with persistent symptomatic lesions following multidisciplinary evaluation.

For patients who fail to achieve an adequate response to propranolol or in whom treatment is contraindicated, alternative therapeutic strategies include surgical excision, laser therapy, and systemic corticosteroids, depending on lesion characteristics and clinical presentation [5].

Herein, we present the case of a 15-year-old girl with a persistent pediatric-onset oropharyngeal hemangioma who developed progressively worsening symptoms of upper airway obstruction. Flexible nasolaryngoscopy demonstrated significant airway compromise, while CT angiography provided comprehensive characterization of the lesion's extent and vascular supply. Following multidisciplinary evaluation, oral propranolol therapy was initiated, and the patient underwent close clinical surveillance by the Otolaryngology-Head and Neck Surgery and Cardiology teams.

## Case presentation

A 15-year-old Hispanic female with a persistent vascular lesion of the soft palate first recognized at seven years of age was referred to the emergency department from a primary care center because of progressive enlargement of the lesion associated with intermittent dyspnea, odynophagia, and globus sensation. She denied spontaneous bleeding or previous treatment for the lesion. Although the late clinical recognition and persistence of the lesion beyond early childhood were atypical for classic infantile hemangioma, no vascular lesion had been documented at birth, and the available clinical history did not allow reliable differentiation among a persistent pediatric-onset hemangioma, congenital hemangioma, or other vascular anomalies.

**Figure 1:**
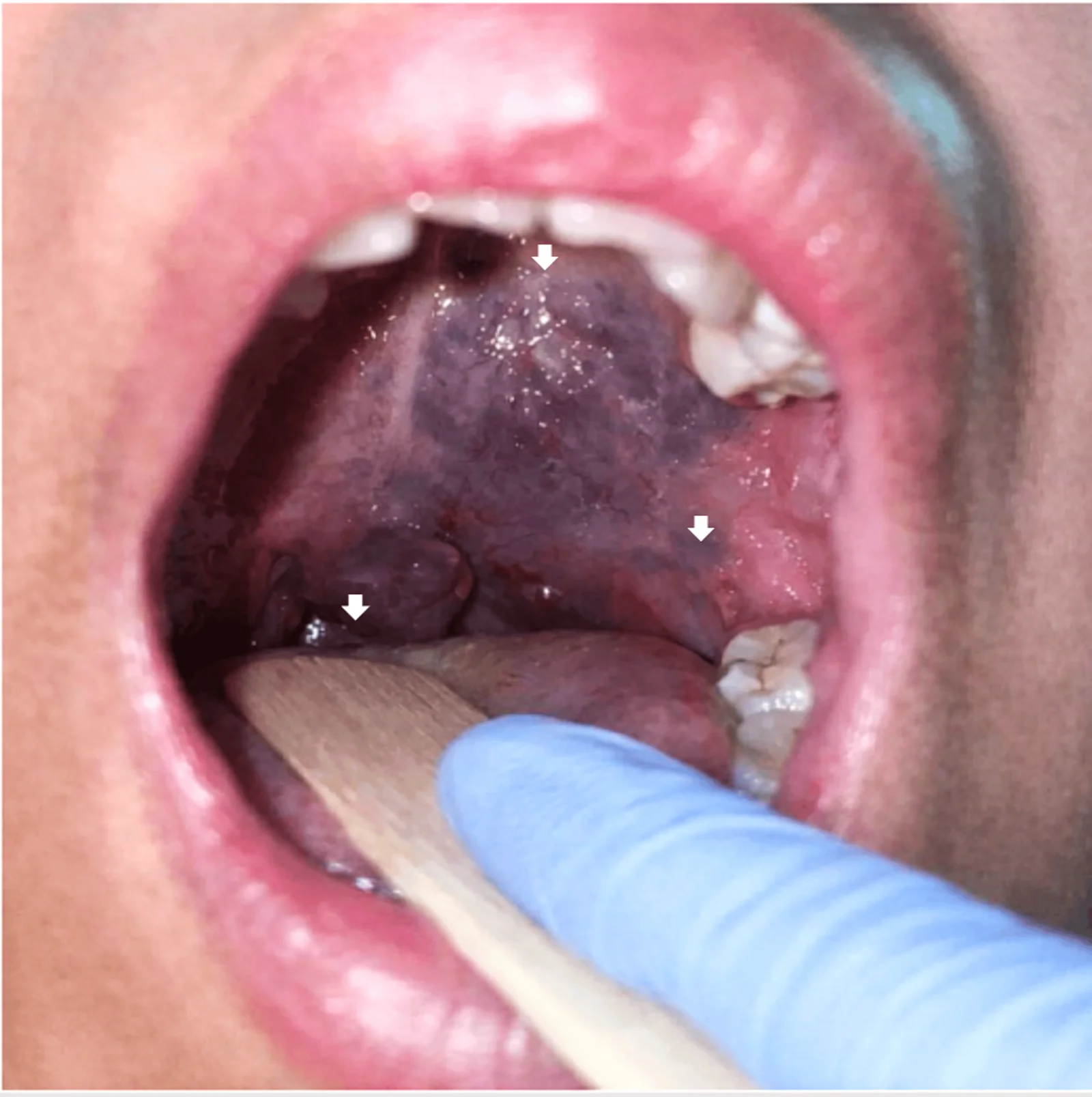
Clinical photograph at presentation Well-circumscribed, non-ulcerated submucosal blue-violet vascular lesion with a smooth surface and subtle lobulation, without evidence of active bleeding. The lesion arises from the left soft palate, extends onto the ipsilateral hard palate, and involves the uvula and ipsilateral anterior tonsillar pillar. The bilateral buccal mucosa and the contralateral soft and hard palate remain uninvolved. White arrows indicate the superior, lateral, and inferior extent of the lesion.

On examination, the patient was hemodynamically stable and maintained normal oxygen saturation for the altitude of Bogotá while breathing room air. She exhibited no stridor, orthopnea, nocturnal respiratory symptoms, or signs of impending airway compromise. Oral examination demonstrated preserved mouth opening and rightward deviation of the uvula with violaceous discoloration. A large violaceous submucosal lesion involving the left soft palate, uvula, left palatine tonsil, posterior tonsillar pillar, and tongue base produced marked narrowing of the oropharyngeal airway (Figure 1). The clinical estimate of approximately 6 cm reflected the maximum surface extension of the lesion observed during physical examination, whereas subsequent cross-sectional imaging measured the deeper enhancing component.

**Figure 2:**
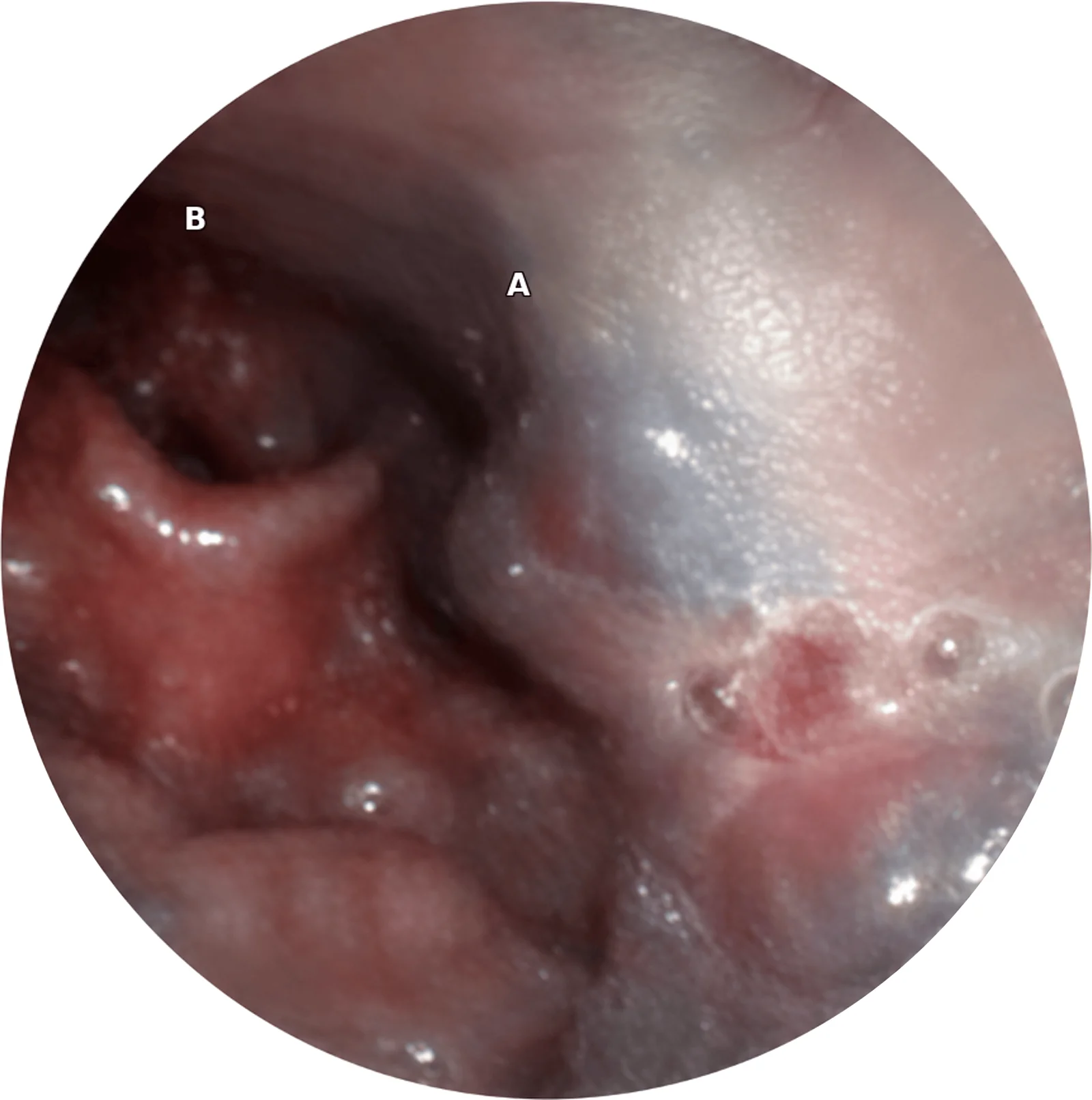
Flexible nasolaryngoscopy at presentation Endoscopic view demonstrating an extensive violaceous submucosal vascular lesion arising from the left soft palate and extending along the left lateral oropharyngeal wall toward the ipsilateral tongue base, resulting in marked narrowing of the oropharyngeal airway while preserving a patent residual lumen. (A) Submucosal vascular lesion. (B) Residual patent oropharyngeal airway lumen. The vallecula and glottis are uninvolved, with no evidence of endolaryngeal extension.

Flexible nasolaryngoscopy (Figure 2) demonstrated an extensive violaceous submucosal lesion involving the left soft palate and lateral oropharyngeal wall, producing significant narrowing of the upper airway without complete obstruction or endolaryngeal extension.

**Figure 3:**
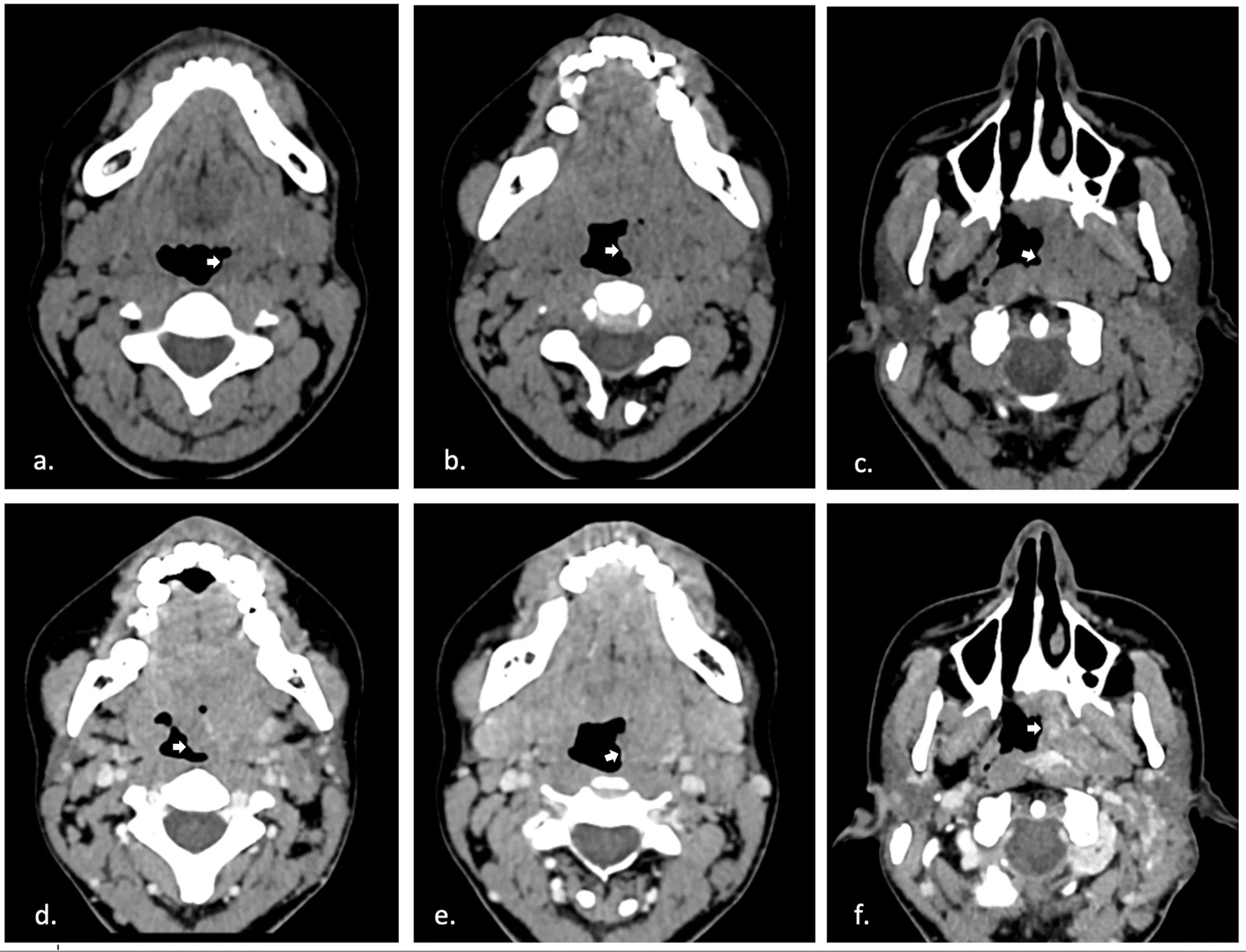
Contrast-enhanced CT Axial non-contrast (a–c) and contrast-enhanced (d–f) computed tomography of the paranasal sinuses and maxillofacial region demonstrates a heterogeneous soft-tissue mass centered within the left pharyngeal mucosal space, extending across the nasopharynx and oropharynx. Following intravenous contrast administration, the lesion exhibits heterogeneous enhancement, consistent with a vascular neoplasm.

**Figure 4:**
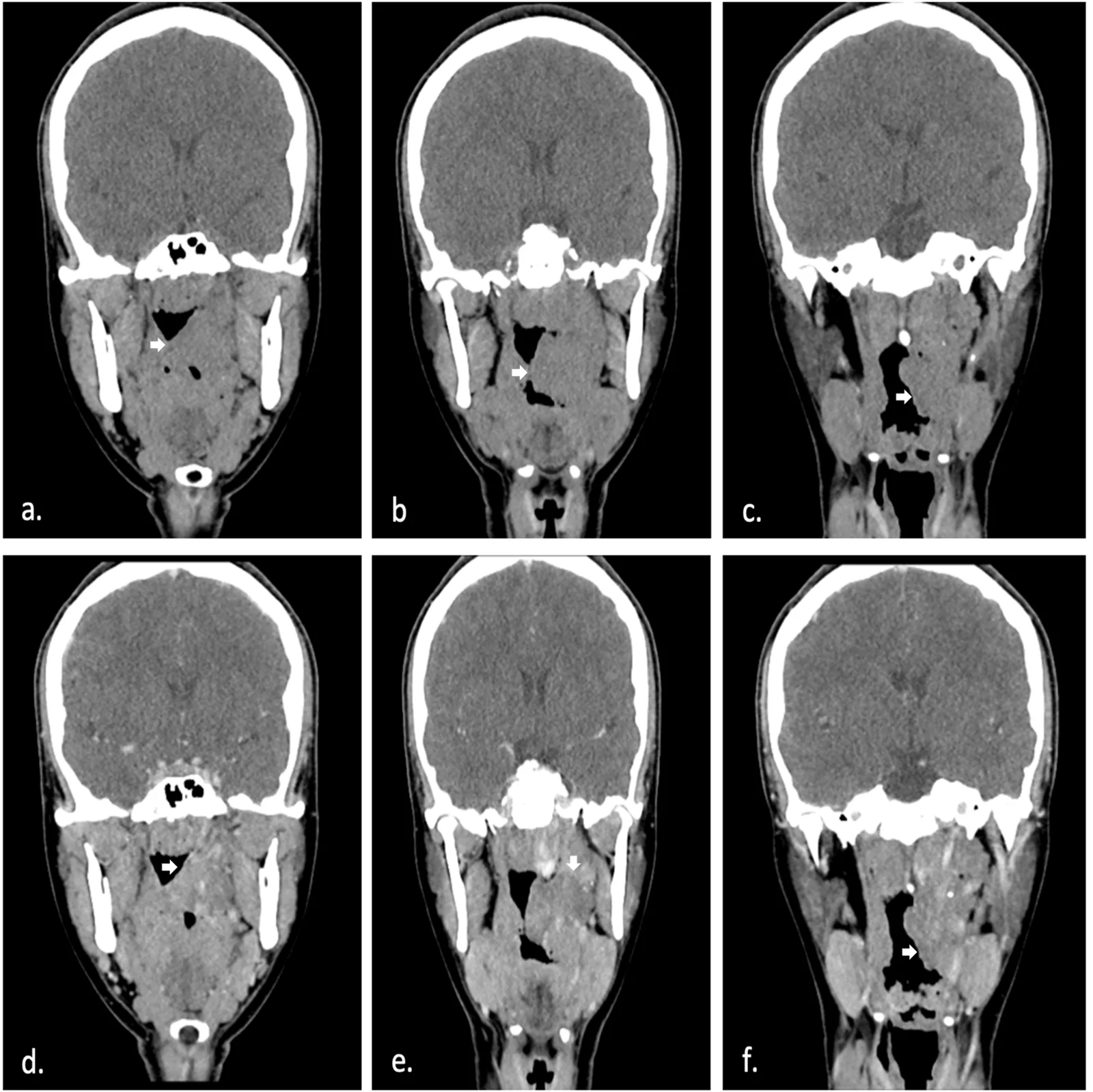
CT angiography Coronal non-contrast (a–c) and contrast-enhanced (d–f) CT angiographic images demonstrate an extensive hypervascular soft-tissue mass centered in the left soft palate, with superior extension into the nasopharynx and inferior extension into the oropharynx. Following intravenous contrast administration, the lesion exhibits heterogeneous enhancement, allowing precise delineation of its anatomical extent and vascular characteristics.

Given the clinical suspicion of a vascular lesion, the patient was admitted for further evaluation. Laboratory investigations, including complete blood count, serum biochemistry, and renal function tests, were within normal limits and revealed no abnormalities that would modify the proposed therapeutic approach. Contrast-enhanced computed tomography demonstrated a heterogeneous enhancing soft-tissue mass measuring 36 × 30 × 40 mm within the left pharyngeal mucosal space without evidence of osseous erosion or invasion of adjacent structures (Figure 3). CT angiography confirmed the hypervascular nature of the lesion while providing detailed characterization of its anatomical extent and vascular supply (Figure 4). Although the imaging findings strongly supported a vascular neoplasm, neither CT nor CT angiography alone could reliably distinguish a hemangioma from other vascular anomalies. Therefore, the diagnosis was considered a probable clinicoradiologic diagnosis based on the combined clinical presentation and imaging findings according to the ISSVA classification. Magnetic resonance imaging, Doppler ultrasonography, and tissue biopsy were not performed.

Following multidisciplinary evaluation by the Otolaryngology-Head and Neck Surgery and Cardiology teams, with joint assessment by the Head and Neck Surgery service, surgical resection was considered to carry an unfavorable risk-benefit profile because of the lesion's extensive involvement of critical oropharyngeal structures and the anticipated functional morbidity. Given the absence of established treatment protocols for persistent pediatric-onset vascular lesions presenting during adolescence, oral propranolol was selected as an individualized therapeutic approach based on its established efficacy in complicated infantile hemangiomas and its more favorable anticipated morbidity compared with surgical intervention. Propranolol was initiated at 0.5 mg/kg/day, administered orally in two divided doses every 12 hours, with close outpatient follow-up by the Otolaryngology-Head and Neck Surgery and Cardiology teams. Baseline cardiovascular evaluation revealed no contraindications to beta-blocker therapy. The patient and her family received detailed counseling regarding warning signs, including worsening dyspnea, bleeding, poor oral intake, syncope, or symptoms suggestive of hypoglycemia, together with instructions to seek immediate medical attention should these occur.

At the six-month follow-up evaluation, the patient reported complete resolution of respiratory symptoms, without bleeding episodes or treatment-related adverse effects. Clinical examination suggested a mild reduction in the apparent volume of the lesion, with no evidence of further enlargement or progression. Because of the marked clinical improvement and satisfaction with the therapeutic outcome, the patient declined repeat flexible nasolaryngoscopy and additional imaging studies. Continued multidisciplinary follow-up was recommended to monitor long-term clinical response and guide future management.

## Discussion

Hemangiomas are benign vascular tumors characterized by endothelial proliferation that may arise in a variety of tissues, including the skin, mucosa, skeletal muscle, glandular tissue, and bone [2]. They account for approximately 7-10% of tumors diagnosed during infancy, with more than half occurring in the head and neck region. However, involvement of the oral cavity remains uncommon, particularly within the soft palate and oropharynx [2]. A marked female predominance has consistently been reported, and recognized risk factors include female sex, multiple gestation, placental abnormalities, family history of infantile hemangioma, prematurity, and low birth weight [8-10].

Infantile hemangiomas typically undergo a proliferative phase during the first year of life, followed by spontaneous involution throughout childhood, whereas congenital hemangiomas are fully developed at birth and either rapidly involute or persist without regression [2]. Consequently, persistent pediatric-onset vascular lesions presenting during adolescence are distinctly uncommon and warrant careful diagnostic evaluation. In the present case, the atypical clinical course, together with the absence of magnetic resonance imaging (MRI), Doppler ultrasonography, and histopathologic confirmation, did not allow definitive biological classification according to the ISSVA framework. Accordingly, the lesion was considered a probable clinicoradiologic diagnosis, while persistent congenital hemangioma, venous malformation, lymphatic malformation, and other vascular tumors remained important diagnostic considerations according to the current ISSVA classification [2,11].

Although the pathogenesis of infantile hemangiomas remains incompletely understood, current evidence supports a multifactorial process involving dysregulated vasculogenesis and angiogenesis, hypoxia-induced endothelial activation, and molecular pathways shared with placental microvasculature [3-5,10,12,13]. From a clinical perspective, however, differentiation from other vascular anomalies is achieved through the integration of clinical history, physical examination, endoscopic evaluation, and imaging findings rather than pathogenetic mechanisms alone [2].

The differential diagnosis of persistent pediatric-onset vascular lesions involving the oropharynx includes congenital hemangioma, venous malformation, lymphatic malformation, hemangioendothelioma, pyogenic granuloma, and other vascular tumors [14,15]. Given the unusual persistence of the lesion into adolescence, comprehensive multidisciplinary evaluation was undertaken. Flexible nasolaryngoscopy, contrast-enhanced computed tomography, and CT angiography provided detailed characterization of the lesion's extent, vascular anatomy, and upper airway involvement, allowing formulation of a probable clinicoradiologic diagnosis and guiding individualized treatment planning based on the overall clinical and radiologic findings [5,8].

Imaging plays a central role in the evaluation of vascular lesions arising in anatomically complex regions, such as the head and neck [2]. Magnetic resonance imaging is generally regarded as the preferred modality for characterizing soft-tissue vascular lesions because of its superior soft-tissue contrast and its ability to define tissue planes [9,16]. In the present case, however, flexible endoscopic assessment combined with contrast-enhanced computed tomography and CT angiography provided comprehensive evaluation of the lesion's extent, vascular architecture, and relationship to adjacent structures, supporting a probable clinicoradiologic diagnosis and appropriate therapeutic decision-making [5,8,17]. Although magnetic resonance imaging and histopathologic examination may provide complementary information for biological classification according to the ISSVA framework, the multimodal assessment performed in this case effectively guided the diagnostic evaluation and individualized multidisciplinary management.

The decision to initiate systemic therapy should be guided primarily by lesion-related complications rather than by the diagnosis alone. Indications include functional impairment, ulceration, hemorrhage, permanent disfigurement, visual compromise, upper airway obstruction, and, in severe cases, high-output cardiac failure [17]. In the present case, progressive airway narrowing associated with intermittent dyspnea and dysphagia justified active treatment despite the patient's atypical age at presentation.

Although propranolol has become the standard first-line therapy for complicated infantile hemangiomas during infancy, evidence supporting its use in persistent pediatric-onset vascular lesions presenting during adolescence remains limited [10]. Therefore, treatment decisions in these uncommon scenarios should be individualized according to multidisciplinary assessment, lesion characteristics, symptom burden, and the anticipated risks and benefits of alternative therapeutic strategies.

Propranolol was selected following multidisciplinary evaluation involving otolaryngology, cardiology, and radiology. Because the lesion extensively involved the soft palate, uvula, anterior tonsillar pillar, and lateral oropharyngeal wall, surgical excision was considered technically challenging and associated with a substantial risk of hemorrhage, postoperative velopharyngeal dysfunction, dysphagia, and speech impairment. Likewise, other interventional approaches, including embolization, laser therapy, and sclerotherapy, were not considered first-line options because of the lesion's diffuse submucosal extension, anatomical complexity, and the absence of immediate life-threatening airway compromise. Consequently, an initial conservative strategy with oral propranolol represented the most appropriate therapeutic approach.

The biological basis for propranolol treatment is well established and includes early vasoconstriction, inhibition of angiogenesis through downregulation of vascular endothelial growth factor (VEGF) and basic fibroblast growth factor (bFGF), and promotion of endothelial apoptosis, ultimately reducing vascular proliferation [8,13,17,18]. In the present case, treatment was initiated at 0.5 mg/kg/day administered in two divided doses following baseline cardiovascular evaluation and exclusion of contraindications. Because the patient demonstrated excellent tolerability and progressive clinical improvement without adverse effects, dose escalation was not considered necessary.

Randomized clinical trials have consistently demonstrated the superiority of propranolol over observation in infants with proliferating infantile hemangiomas [6,13]. Nevertheless, these findings should be interpreted cautiously when extrapolated to persistent pediatric-onset vascular lesions presenting beyond childhood, where high-quality evidence remains limited. In the present case, therapeutic success was defined by complete resolution of respiratory symptoms, absence of bleeding or lesion progression, excellent tolerability, and an apparent mild reduction in lesion volume during clinical follow-up rather than complete radiologic resolution.

At the six-month follow-up visit, the patient remained asymptomatic, reported no episodes of bleeding or respiratory compromise, and experienced no adverse effects related to propranolol therapy. Clinical examination suggested a mild reduction in lesion volume without evidence of progression. Repeat flexible nasolaryngoscopy and imaging were recommended to objectively reassess the lesion; however, the patient declined additional investigations because of sustained clinical improvement. Despite the absence of repeat imaging, the favorable clinical evolution supported continuation of conservative management with close multidisciplinary follow-up.

This case underscores the diagnostic and therapeutic challenges posed by persistent pediatric-onset vascular lesions involving the upper aerodigestive tract. Comprehensive clinical evaluation, flexible endoscopy, contrast-enhanced computed tomography, CT angiography, and multidisciplinary assessment enabled formulation of a probable clinicoradiologic diagnosis and guided individualized management. Although magnetic resonance imaging, Doppler ultrasonography, and histopathologic examination could have provided complementary information for definitive biological classification according to the ISSVA framework, the multimodal evaluation performed in this case supported appropriate therapeutic decision-making. Furthermore, this case illustrates that carefully selected adolescents with symptomatic persistent pediatric-onset vascular lesions may achieve favorable short-term clinical outcomes with conservative treatment when the anticipated morbidity of surgical intervention outweighs its potential benefits. Further studies are warranted to better define the role of conservative management in similarly uncommon clinical scenarios.

## Conclusions

Hemangiomas involving the soft palate with extension to the oropharynx represent an uncommon yet clinically significant entity because of their potential to cause progressive upper airway compromise. This case underscores the importance of a meticulous diagnostic approach integrating clinical assessment, endoscopic examination, and advanced vascular imaging to accurately define lesion extent, characterize its vascular anatomy, and support a probable clinicoradiologic diagnosis, thereby guiding individualized therapeutic decision-making.

Following multidisciplinary evaluation, oral propranolol was initiated because the anticipated morbidity of surgical resection outweighed its potential benefits. During six months of clinical follow-up, the patient demonstrated resolution of respiratory symptoms, no treatment-related adverse effects, and apparent mild reduction in lesion volume, although repeat endoscopic and radiologic evaluation was declined. This case contributes to the current understanding of persistent pediatric-onset vascular lesions presenting in anatomically critical locations during adolescence and highlights the importance of individualized multidisciplinary management. Further studies are warranted to better define optimal diagnostic and therapeutic strategies for these uncommon clinical presentations.
